# Supplementary material for: Reversed asymmetric warming of sub-diurnal temperature over land during recent decades
Source: Nat Commun. 2023 Nov 8;14:7189. doi: 10.1038/s41467-023-43007-6 (PMC10632450; doi:10.1038/s41467-023-43007-6)
Supplement: Supplementary file 1 — Supplementary Information [file 41467_2023_43007_MOESM1_ESM.pdf]

Supplementary Information for

Reversed asymmetric warming of sub-diurnal temperature over land during recent decades

Ziqian Zhong[1,2], Bin He[1,*], Hans W. Chen[2], Deliang Chen[3], Tianjun Zhou[4], Wenjie Dong[5], Cunde Xiao[6], Shang-ping Xie[7], Xiangzhou Song[8], Lanlan Guo[6], Ruiqiang Ding[6], Lixia Zhang[4], Ling Huang[9], Wenping Yuan[5], Xingming Hao[10], Duoying Ji[1], Xiang Zhao[11]

[1]State Key Laboratory of Earth Surface Processes and Resource Ecology, Beijing Normal University, Beijing 100875, China.

[2]Department of Space, Earth and Environment, Division of Geoscience and Remote Sensing, Chalmers University of Technology, SE-412 96 Gothenburg, Sweden

[3]Regional Climate Group, Department of Earth Sciences, University of Gothenburg, S-40530 Gothenburg, Sweden.

[4]Institute of Atmospheric Physics, Chinese Academy of Sciences, Beijing 100029, China

[5]School of Atmospheric Sciences, Sun Yat-Sen University, Guangzhou 510275, China.

[6]State Key Laboratory of Earth Surface Processes and Resource Ecology, School of Geography, Beijing Normal University, Beijing 100875, China

[7]Scripps Institution of Oceanography, University of California San Diego, La Jolla, CA 92039, USA.

[8]Key Laboratory of Marine Hazards Forecasting, Ministry of Natural Resources (MNR), Hohai University, Nanjing 210024, China

[9]College of Urban and Environmental Sciences, Peking University, Beijing 100871, China

[10]State Key Laboratory of Desert and Oasis Ecology, Xinjiang Institute of Ecology and Geography, Chinese Academy of Sciences, Urumqi 830011, China.

[11]State Key Laboratory of Remote Sensing Science, Beijing Normal University, Beijing 100875, China

*e-mail: hebin@bnu.edu.cn

This PDF file includes:

Supplementary Fig. 1 to 10

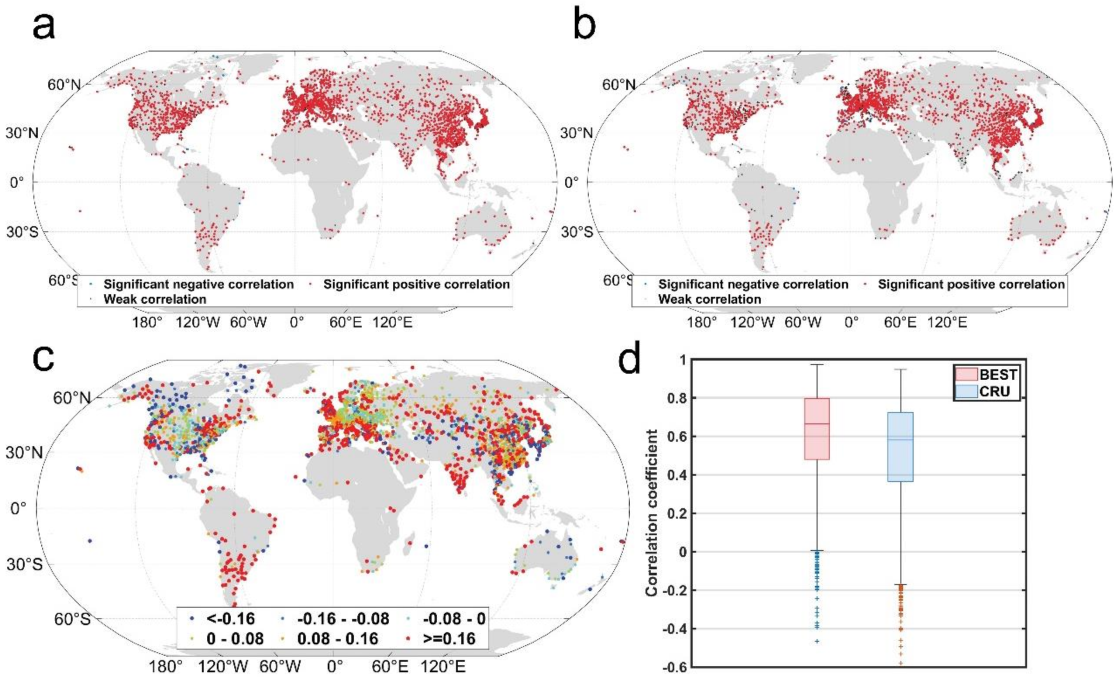

Supplementary Fig 1. Spatial distribution of the relationship between Global Surface Summary of the Day (GSOD) diurnal temperature range (DTR) observations and BEST DTR (a), as well as CRU TS DTR (b), based on Pearson correlation analysis. (c) Spatial distribution of the difference between the Pearson correlation coefficient of GSOD DTR observations and BEST DTR, and the Pearson correlation coefficient of GSOD temperature observations and CRU TS DTR. (d) Boxplot visualization of the Pearson correlation coefficient for GSOD DTR observations with both BEST DTR and CRU TS DTR. The height of each box indicates the interquartile range, the notch of each box indicates the median, and the bottom and top of the box indicate the first and third quartiles, respectively. The whiskers extend to the most extreme regression coefficient. The outliers, which is a value that is more than 1.5 times the interquartile range away from the bottom or top of the box, are plotted individually using the '+' marker symbol. All correlation analyses were performed for the period 1978-2020.

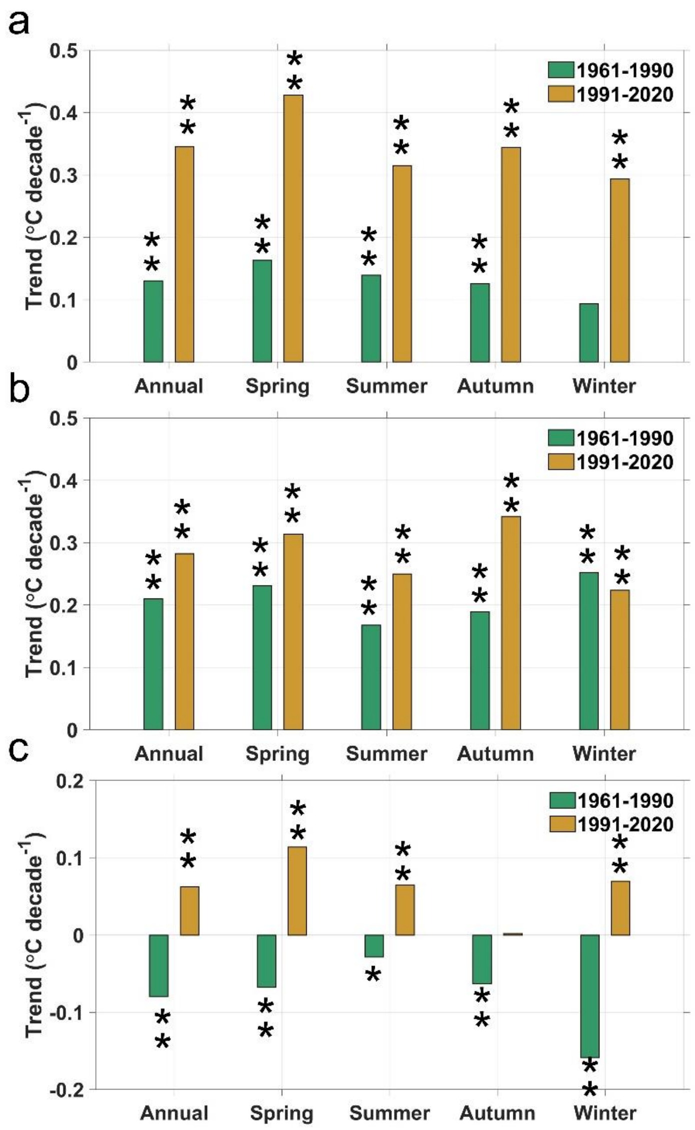

Supplementary Fig 2. Annual and seasonal trends in global mean land surface maximum temperature (a), minimum temperature (b), and diurnal temperature range (c) during 1961-1990 (green bar) and 1991-2020 (orange bar). The asterisk indicates the significant level (*: p<0.1, **: p<0.05). The temperature was extracted from the BEST dataset.

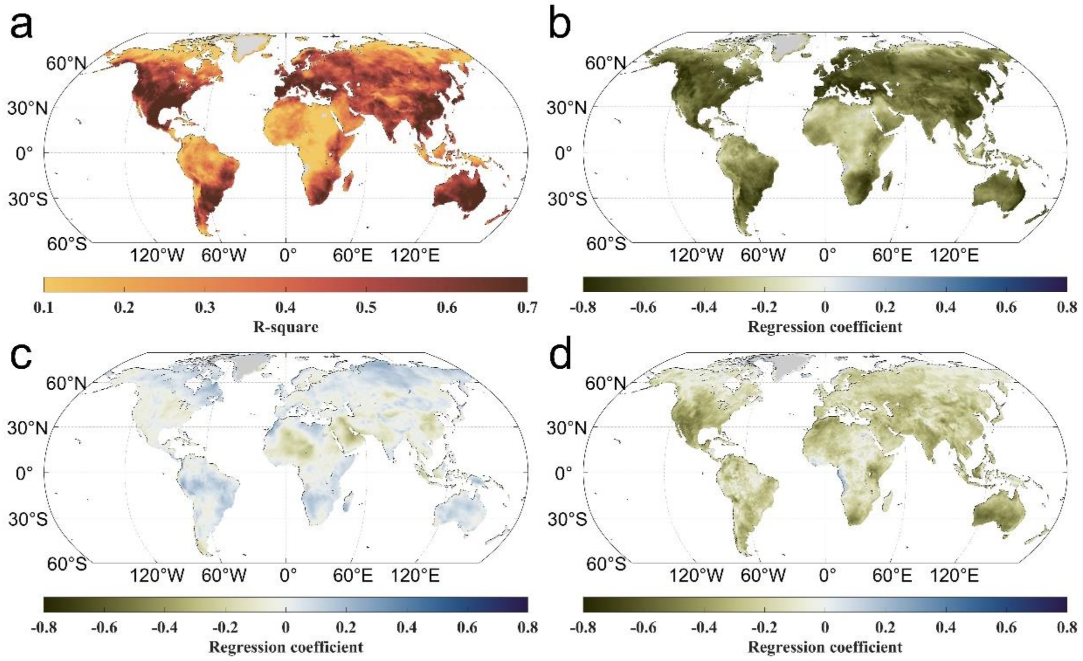

Supplementary Fig 3. (a) Evaluation of the ridge regression model using determination coefficients in the validation dataset, along with the spatial distribution of regression coefficients for total cloud cover (b), aerosol optical depth (c), and soil moisture (d) with respect to diurnal temperature range (DTR) during 1981-2020. The ridge regression analysis was performed by randomly dividing the original anomalies into an 80% calibration dataset and a 20% validation dataset. The training set dataset was used to train the ridge regression model, while the validation set dataset was employed to evaluate its performance. Subsequently, all the datasets were merged to determine the ridge regression coefficients. Only grid cells exhibiting regression results that passed the significance test ($p<0.05$) are shown. The total cloud cover was from ERA5 dataset, aerosol optical depth was from MERRA-2, and soil moisture was from GLEAM dataset.

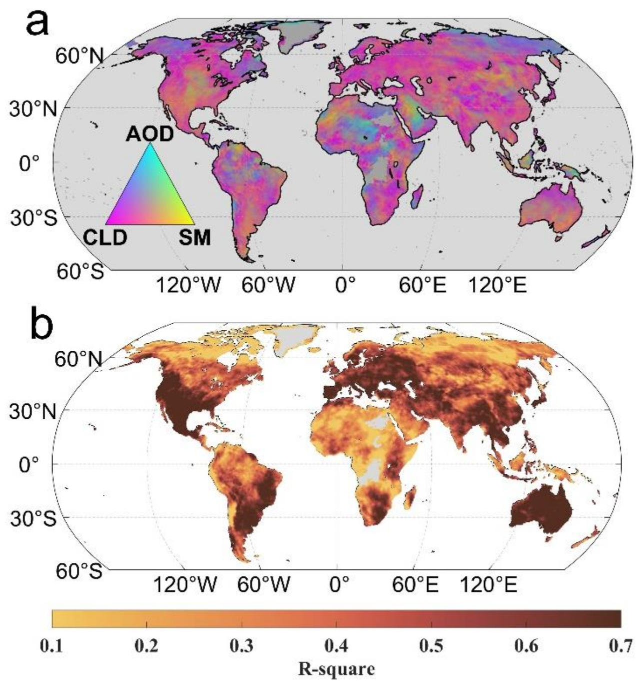

Supplementary Fig 4. (a) The contributions of total cloud cover (CLD; magenta), aerosol optical depth (AOD; cyan) and soil moisture (SM; yellow) to diurnal temperature range (DTR) changes during 2003–2020. Here the total cloud cover is derived from the Moderate Resolution Imaging Spectroradiometer (MODIS) dataset. The color of the composite was determined by the relative contribution from the magnitude of the ridge regression coefficients. (b) Spatial distribution of the determination coefficients (R^2) of the regression model. Only the grid cells with the regression result that passed the test of significance ($p<0.05$) in the training set are shown. The AOD was from MERRA-2, and SM was from GLEAM dataset.

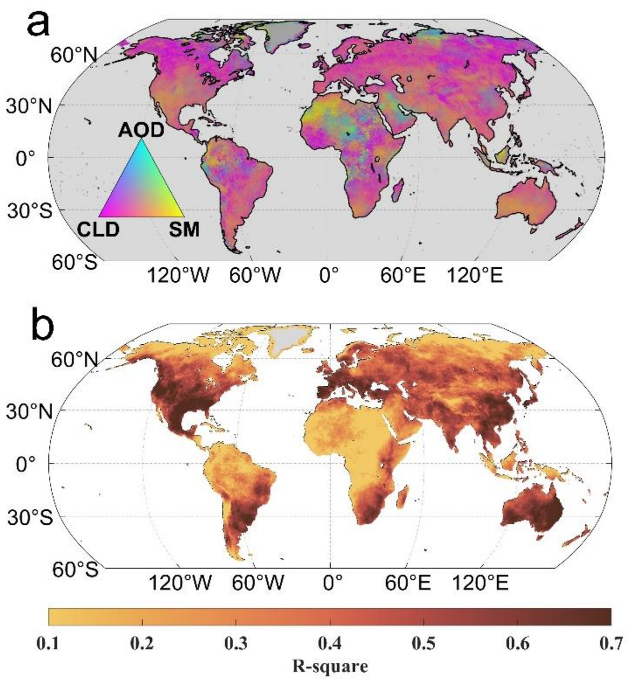

Supplementary Fig 5. (a) The contributions of total cloud cover (CLD; magenta), aerosol optical depth (AOD; cyan) and soil moisture (SM; yellow) to diurnal temperature range (DTR) changes during 1981–2020. Here the map composite was determined based on the relative importance of predictor variables for predicting errors, which were derived using the Random Forest algorithm. (b) Spatial distribution of the determination coefficients (R^2) of the regression model. The CLD was from the fifth-generation ECMWF reanalysis (ERA5) dataset, AOD was from MERRA-2, and SM was from GLEAM dataset.

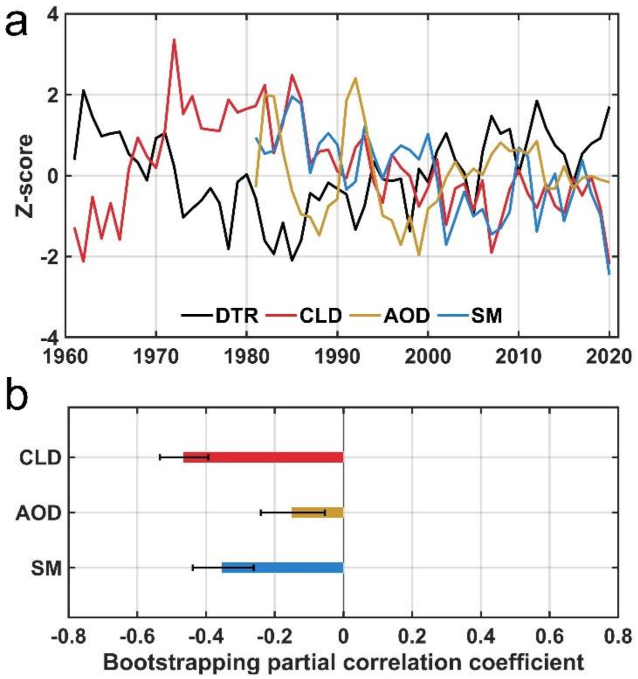

Supplementary Fig 6. (a) Global area-weighted annual average anomalies of diurnal temperature range (DTR), total cloud cover (CLD), aerosol optical depth (AOD), and soil moisture (SM). The z-score anomalies were calculated using monthly climatology (from 1981 to 2020) means and standard deviations. (b) Bootstrapped partial correlation coefficients between annual DTR and CLD, AOD, and SM during 1981-2020. The mean bootstrapped partial correlation coefficients between annual DTR and CLD, AOD, and SM are -0.47 (-0.54 to -0.41, 95% CI), -0.18 (-0.28 to -0.07, 95% CI) and -0.31 (-0.39 to -0.23, 95% CI), respectively. The error bars represent the range from the 0.5th to the 99.5th percentile. When conducting partial correlation analysis between DTR and one environmental variable, the influence of the other two environmental variables was controlled. The DTR temperature and CLD data here is sourced from the ERA5 dataset, AOD was from MERRA-2, and SM was from the GLEAM dataset.

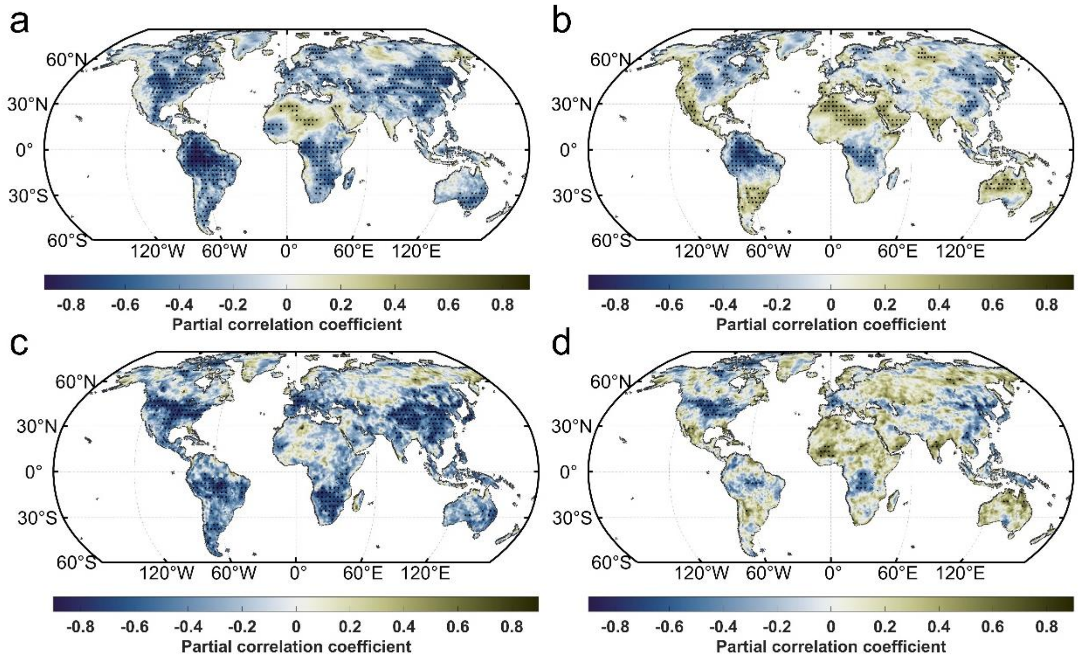

Supplementary Fig 7. Spatial distribution of partial correlation coefficients between total cloud cover and maximum temperature (a) or minimum temperature (b) during 1981-2020, while controlling for the influence of soil moisture. The black dots mark the areas where correlations are significant at the $p < 0.05$ level. The total cloud cover was obtained from ERA5 dataset. (c) to (d), same as (a) to (b), but the total cloud cover was obtained from MODIS (2003-2020) dataset.

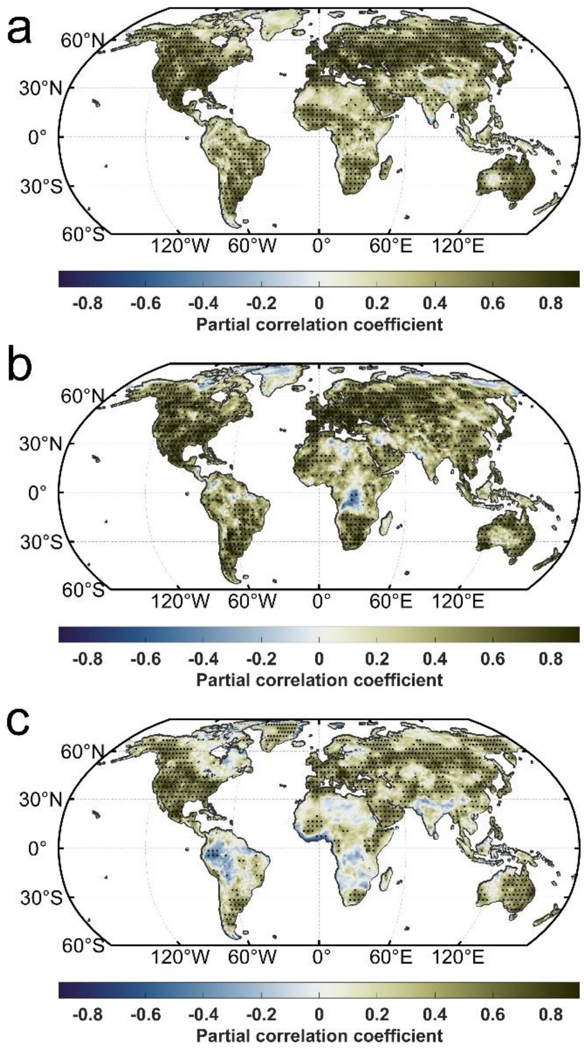

Supplementary Fig 8. The spatial distribution of partial correlation coefficients between diurnal temperature range (DTR) and incident shortwave radiation, while controlling for the influence of soil moisture. The incident shortwave radiation dataset was obtained from ERA5 (1981-2020, a), CERES (2001-2020, b) and MERRA-2 (1981-2020, c), respectively. The black dots mark the areas where correlations are statistically significant at the $p < 0.05$ level.

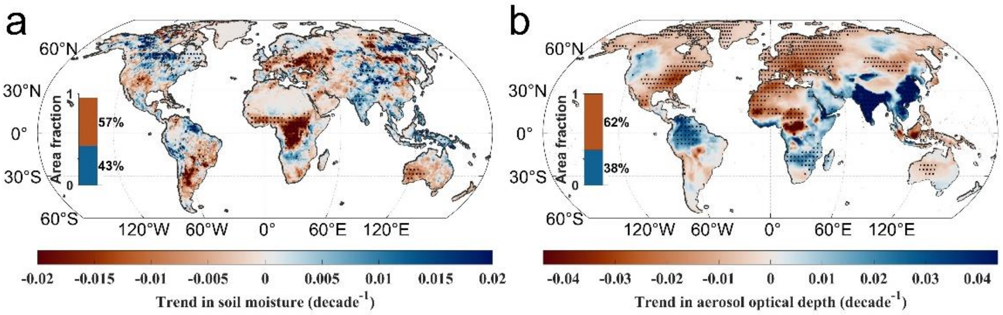

Supplementary Fig 9. Spatial distribution of trends in (a) soil moisture and (b) aerosol optical depth during 1991-2020. The black dots mark the areas where changes are statistically significant at the $p < 0.05$ level. The aerosol optical depth was from MERRA-2, and and soil moisture was from GLEAM dataset.

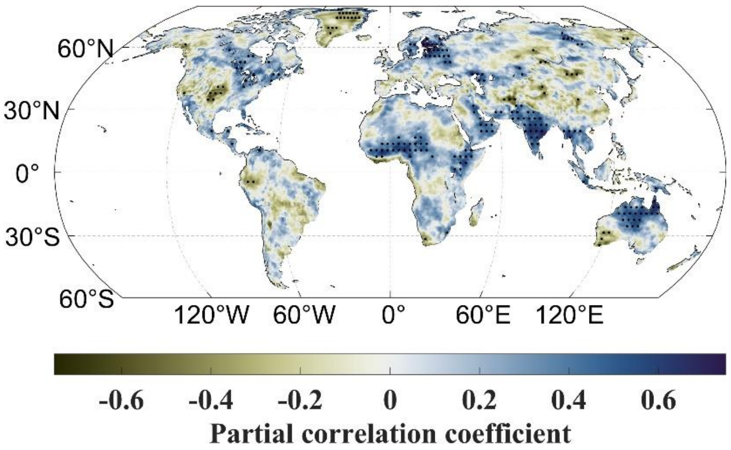

Supplementary Fig 10. Spatial distribution of partial correlation coefficient between annual BEST diurnal temperature range (DTR) and surface albedo during 1981-2020, control the effect of total cloud cover, aerosol and soil moisture. The black dots mark the areas where correlations are significant at the $p < 0.05$ level. The surface albedo was obtained from MERRA-2 dataset.
